# Conversion Disorder: Early Diagnosis and Personalized Therapy Plan Is the Key

**DOI:** 10.1155/2020/1967581

**Published:** 2020-01-10

**Authors:** Lauren Miller, Robert L. Archer, Nidhi Kapoor

**Affiliations:** Department of Neurology, University of Arkansas for Medical Sciences, Little Rock, AR, USA

## Abstract

Conversion disorder is characterized by one or more symptoms of altered voluntary motor or sensory functions that cannot be explained by a neurological disease (Keynejad, 2019; Samuels et al., 2019). We present a patient with conversion disorder and discuss her process in overcoming this disorder. Additionally, we review the literature about this specific disorder. A 15-year-old white female was diagnosed with conversion disorder and has shown significant recovery with physical therapy and group therapy since. It is essential to recognize this disorder early to lessen the financial burden on families and to speed up the recovery process for these patients.

## 1. Introduction

Conversion disorder, formerly known as hysteria, can be defined as one or more symptoms of altered voluntary motor or sensory functions that cannot be explained by a neurological disease [1, 2]. Conversion disorder is usually seen in women and younger aged people, but rare before age 10 and often clinical based [3]. Often this disorder is caused by childhood trauma, a stressful event, physical and sexual abuse, and depression/anxiety [4].

## 2. Case Report

A 15-year-old white female presented with flu-like symptoms, progressed 2 days later to throwing up blood. She was sent to the Emergency Room where she was diagnosed with possible appendicitis and was discharged the next day. That weekend, she was still vomiting, unable to hold food down, and nauseated. The patient was admitted to Arkansas Children's Hospital (ACH) for 1–2 weeks where she received extensive workup including MRI, CT scan, gastrointestinal imaging, and a spinal tap, and all the results came back normal. She was also evaluated by an ophthalmologist and a cardiologist, and no organic etiology was found for her symptoms. She was discharged home with an unknown diagnosis and started on symptomatic medical management to help relieve some of the symptoms. Soon after discharged from ACH, her legs started getting weak, and it was discussed that she may be having some neurological disorders and would need to be evaluated further. Presenting with symptoms of throwing up, unable to keep food down, leg numbness, and a constant migraine, she was readmitted at ACH for another two weeks where even more extensive workup was done again, including CT and MRI scans of the brain. All results came back normal, and she was sent home without a true diagnosis and was treated for depression and anxiety.

She was referred to a neurologist at the University of Arkansas for Medical Sciences (UAMS) 2–3 weeks later where she was evaluated and admitted to the hospital for one day to treat migraine. With no evidence of any organic etiology found on the extensive workup and no sign of relief with continued treatment, she was diagnosed with conversion disorder. She was discharged to receive physical therapy (3x a week) and cognitive behavioral therapy (1x a week) and also seen by a therapist. The therapist uncovered that from the age of 6–12 years, the patient experienced two active addicted parents, and she received physical and mental abuse from her father. During that time, she was required to become the head of the household to ensure bills were being paid and that her parents were being taken care of. At the age of 12, her mom received de-addiction treatment for 3–4 months and has been clean and sober for the past 5 years. Between the ages of 12 and 13, her parents got divorced, and she moved with her mom. She attended high school for one year but then transferred to another city. Growing up in an environment where she was in a constant fight-or-flight mode, she found a sense of security at this new city and was able to relax. The February of her sophomore year, she started gradually showing signs of weakness on the right side, seizures (nonepileptic spells), tingling, and numbness in her legs. Seven months later, she was referred and accepted into Mayo Clinic as outpatient for further management. At Mayo Clinic, she went to group therapy everyday from 8 am to 4 pm for 4 weeks with people who were experiencing other neurological disorders not just conversion disorder. During the group therapy at Mayo Clinic, she not only went through physical therapy and occupational therapy but also learned how to stay on a schedule, how to live a normal life with conversion disorder, and how to manage and tolerate the pain. She believes that the group therapy at Mayo helped her the best in her recovery.

The patient states that there were no real triggers that caused this disorder, but her history could have been an underlying cause. The patient is now still in recovery, all symptoms have improved but not completely gone away. The patient still experiences numbness and weakness on the right side, headaches, nausea, and pain in the right leg. She learned, at Mayo Clinic, how to tolerate and deal with the pain, which she believes helps her. The patient is currently on Topamax, iron, vitamin D3, and calcium. The patient plans to graduate high school this year and plans to attend college as premedical.

## 3. Discussion

### 3.1. Diagnosis

Conversion disorder is hard to diagnose due to symptoms being similar to other disorders. One key factor in identifying conversion disorder is its sudden onset of symptoms including nonepileptic seizure, weakness, and paralysis; abnormal movement; visual, speech, and sensory loss; or finally globus sensation [5]. Several tests can be done to ensure that the diagnosis of conversion disorder is correct [5]. Those tests include Hoover sign, fingertip test, signature test, menace reflect, tearing reflex, optokinetic test, cocontracting sign, and finally sternocleidomastoid test [5].

### 3.2. Misdiagnosis

Since there is little research on conversion disorder, it can often go misdiagnosed, which can lead to patients spending even more money for tests and treatment that might not relieve symptoms. Some common misdiagnoses are multiple sclerosis (MS), stroke or a spinal disorder, myasthenia gravis, movement disorder, epilepsy, laryngeal dystonia, factitious disorder, or malingering [6]. People with these disorders, when compared to people with conversion disorder, have different outcomes. For instance, a person with myasthenia gravis will show bulbar symptoms, diplopia, ptosis, and may have autoantibodies while a patient with conversion disorder will not have these symptoms instead they will have definite evidence of inconsistent movement without fatigue [6].

### 3.3. Prognosis

In patients with conversion disorder, early diagnosis often leads to good prognosis and is crucial to having a successful outcome. Once a physician confirms that a patient's symptoms do not conform anatomically and are atypical or do not correlate with physical examination or neurological finding, they should immediately be referred to a psychiatrist or psychologist for further assessment including an assessment regarding psychiatric comorbidities [7]. Furthermore, the patient should be given a treatment plan designed personally for them based on their symptoms and prognosis of the disorder [7].

### 3.4. Treatment

When presenting a treatment plan to someone with conversion disorder, four levels can make the process easier. First is to educate the patient; provide a diagnosis, discuss why you made that decision, and emphasize that the patient does not have a neurological disease [8]. Secondly, a physician needs to focus on what type of therapy would be best in relieving their symptoms [8]. Some of the most common therapies seen to help people with conversion disorder are physical therapy, group therapy, or family therapy, or even in some cases, a placebo effect was seen useful [8]. Thirdly, if standard therapy still does not relieve symptoms, they can be treated with psychotherapies, which include pharmacotherapy, psychodynamic psychotherapy, hypnosis, and so forth [8]. Lastly, when all other resources have been exerted, you can treat a patient with transcranial magnetic stimulation (TMS), sedation, and biofeedback [8].

## 4. Conclusion

Conversion disorder is more common than people think, but due to lack of research and knowledge of how to properly diagnose a patient, it oftentimes gets misdiagnosed. This disorder is debilitating to the patient and can be extremely costly if not diagnosed early. Therefore, prompt recognition and treatment stand to help significantly reduce patient morbidity and the health care cost.

## References

[1] Keynejad R. C., Frodl T., Kanaan R., Pariante C., Reuber M., Nicholson T. R. (2019). Stress and functional neurological disorders: mechanistic insights. *Journal of Neurology, Neurosurgery & Psychiatry*.

[2] Samuels A., Tuvia T., Patterson D., Briklin O., Shaffer S., Walker A. (2019). Characteristics of conversion disorder in an urban academic children’s medical center. *Clinical Pediatrics*.

[3] Stone J. F., Sharpe M. (2017). Conversion disorder in adults: epidemiology, pathogenesis, and prognosis. https://www-uptodate-com.libproxy.uams.edu/contents/conversion-disorder-in-adults-epidemiology-pathogenesis-and-prognosis?search=conversion%20disorder&source=search_result&selectedTitle=4~54&usage_type=default&display_rank=4.

[4] Mayo Clinic, Functional neurologic disorders/conversion disorder, https://www.mayoclinic.org/diseases-conditions/conversion-disorder/symptoms-causes/syc-20355197

[5] Stone J., Sharpe M. (2018). *Conversion Disorder in Adults: Clinical Features, Assessment, and Comorbidity*.

[6] Stone J., Sharpe M. (2019). *Conversion Disorder in Adults: Terminology, Diagnosis, and Differential Diagnosis*.

[7] Consultant360 (2014). Sudden vision loss: a case report and overview of conversion disorder. *Consultant360*.

[8] Stone J., Sharpe M. (2018). *Conversion Disorder in Adults: Treatment*.

